# A multicenter study to evaluate the analytical precision by pathologists using the Aperio GT 450 DX

**DOI:** 10.1016/j.jpi.2024.100401

**Published:** 2024-10-09

**Authors:** Thomas W. Bauer, Matthew G. Hanna, Kelly D. Smith, S. Joseph Sirintrapun, Meera R. Hameed, Deepti Reddi, Bernard S. Chang, Orly Ardon, Xiaozhi Zhou, Jenny V. Lewis, Shubham Dayal, Joseph Chiweshe, David Ferber, Aysegul Ergin Sutcu, Michael White

**Affiliations:** a Department of Pathology and Laboratory Medicine, Hospital for Special Surgery, New York, NY, United States of America; b Department of Pathology and Laboratory Medicine, Memorial Sloan Kettering Cancer Center, 1275 York Ave, New York, NY 10065, USA; c Department of Laboratory Medicine and Pathology, University of Washington, School of Medicine (UW) 1959 NE Pacific St., 2nd Floor, Seattle, WA 98195, USA; d Scripps Health, La Jolla, CA, USA; e Global Clinical Affairs, Leica Biosystems Imaging, Inc., Buffalo Grove, IL, USA; f Medical and Scientific Affairs, Leica Biosystems Imaging, Inc., Buffalo Grove, IL, USA

**Keywords:** Aperio GT 450 DX, Digital pathology, Whole slide imaging, Analytical precision

## Abstract

**Background:**

Digital pathology systems (DPS) are emerging as capable technologies for clinical practice. Studies have analyzed pathologists' diagnostic concordance by comparing reviews of whole slide images (WSIs) to glass slides (e.g., accuracy). This observational study evaluated the reproducibility of pathologists' diagnostic reviews using the Aperio GT 450 DX under slightly different conditions (precision).

**Method:**

Diagnostic precision was tested in three conditions: intra-system (within systems), inter-system/site (between systems/sites), and intra- and inter-pathologist (within and between pathologists). A total of five study/reading pathologists (one pathologist each for intra-system, inter-system/site, and three for intra-pathologist/inter-pathologist analyses) were assigned to the respective sub-studies.

A panel of 69 glass slides with 23 unique histological features was used to evaluate the WSI system's precision. Each glass slide was scanned to generate a unique WSI. From each WSI, the field of view (FOV) was generated (at least 2 FOVs/WSI), which included the selected features (1–3 features/FOV). Each pathologist reviewed the digital slides and identified which morphological features, if any, were present in each defined FOV. To minimize recall bias, an additional 12 wild card slides from different organ types were used for which FOVs were extracted. The pathologists also read these wild card slides FOVs; however, the corresponding feature identification was not included in the final data analysis.

**Results:**

Each measured endpoint met the pre-defined acceptance criteria of the lower bound of the 95% confidence interval (CI) overall agreement (OA) rate being ≥85% for each sub-study. The lower bound of the 95% CI for the intra-system OA rate was 95.8%; for inter-system analysis, it was 94.9%; for intra-pathologist analysis, 92.4%, whereas for inter-pathologist analyses, the lower bound of the 95% CI of the OA was 90.6%.

**Conclusion:**

The study results indicate that pathologists using the Aperio GT 450 DX WSI system can precisely identify histological features that may be required for accurately diagnosing anatomic pathology cases.

## Background

Whole slide imaging (WSI) systems allow digitization of glass slides and generate paired WSIs. Digital pathology systems (DPS), including the WSI device (e.g., whole slide scanner), viewer, and monitor, can be used by pathologists to review patient tissue and render histopathology diagnoses. The DPS allows pathologists to review WSI using viewing software and the corresponding display screen and software similar to how glass slides are reviewed on a microscope. DPS has been used for educational and teaching purposes in hospitals and other academic institutions, but with increasing evidence of WSI non-inferiority to traditional microscopy, systems are now being used for diagnostic purposes as well.^1^, ^2^, ^3^, ^4^, ^5^, ^6^, ^7^ One of the essential aspects of anatomic pathology diagnosis is for the pathologist to correctly identify the relevant morphological features of the patient tissue. Very few studies have comprehensively tested the WSI system precision (repeatability and reproducibility) using datasets within/between pathologists and within/between systems.^8^ The current study tested the ability of a group of pathologists using the Aperio GT 450 DX to precisely identify characteristic features of a tissue sample that are required for accurate primary diagnosis.

The Digital Pathology Association (DPA) identified examples of histological features, such as adipocytes, osteoclasts, granulomas, etc., that could be needed for accurate diagnosis. Therefore, identification of these features by a pathologist would increase the possibility of correct precise diagnosis.^8^, ^9^, ^10^

In this study, precision was quantified by evaluating the ability of pathologists to correctly identify these tissue features by measuring repeatability (intra-system and intra-observer) and reproducibility (inter-system and inter-observer) capabilities. The repeatability and reproducibility were estimated by performing three sub-studies, including intra-system (repeatability), inter-system/site (reproducibility), and intra- (repeatability)/inter-pathologist (reproducibility) non-interventional observational assessments. A priori endpoints included a predefined acceptance criteria of 95% confidence interval (CI) lower bound overall agreement (OA) rate being ≥85%.

## Method

### Aperio GT 450 DX

The Aperio GT 450 DX is a WSI system and is composed of the Aperio GT 450 DX scanner, Aperio WebViewer DX software for viewing ScanScope Virtual Slide format image, and a U.S. Food and Drug Administration (FDA) cleared display monitor. Recommended monitors include the Barco MDPC-8127, Dell UP3017, Dell U3023E, and Dell U322QE. The system can scan images at 40× magnification. The WSI system is classified as a Class II and Class A medical device in the U.S. and E.U., respectively.

### Precision panel

A precision panel containing 69 unique formalin-fixed paraffin-embedded (FFPE) hematoxylin and eosin (H&E)-stained samples mounted on a glass slide was used to evaluate precision in identifying the selected histopathological features. The characteristic features were chosen based on the DPA and the FDA recommendations and were the same as used in the previous Aperio AT2 DX System precision study.^8^ Each feature was selected from three different organ sources to have a varied mix of tissue types rather than having a relatively easy-to-identify feature from a single organ source.

**Fig. 1:**
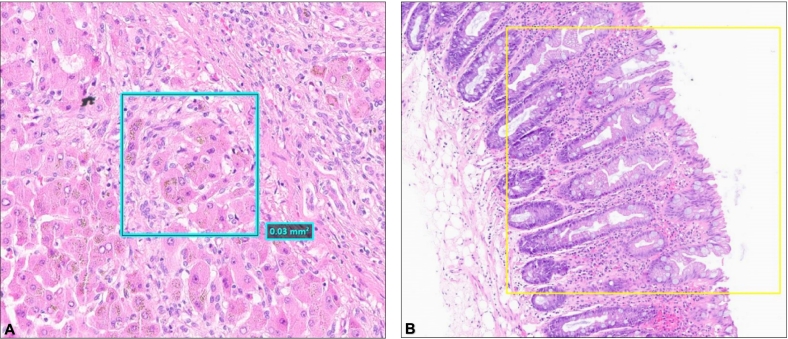
Examples of extracted FOVs. (A) A FOV containing intracellular granules of hemosiderin, and (b) an example of serrated epithelium. The inset box in each shows the FOV specified by the Medical Director/Curating Pathologist.

The curator annotated the features (examples of selected field of view (FOV) features are provided in Fig. 1), which served as the ground truth. The slides chosen from the laboratory information system (LIS) were selected that best represented the designated features in that database and did not necessarily define a perfect morphological characteristic of the selected feature. For example, the study curator/enrollment pathologist screened for Reed-Sternberg cells by searching for “Hodgkin lymphoma”, and selected slides from three different organ sites of the available cases, rather than screening numerous cases of Hodgkin lymphoma to select “classic examples” of Reed–Sternberg cells.

The study curator enrolled all those slides that contained at least one selected histological feature, irrespective of the presence of other secondary features. The slide curation was performed in the previous Aperio AT2 DX System precision study.^8^ For the current study, the curator only quality-checked the slides per the study inclusion/exclusion criteria (as described in the study design section). The curation pathologist did not participate with the reading pathologist team that read the FOV in this observational study.

Based on the selected study feature, each glass slide was scanned at either 20× (12 features extracted from 36 slides) or 40× (11 features extracted from 33 slides) magnification to create a 69-slide panel in the previous Aperio AT2 DX System study.^8^ The same 69-slide panel was used for this study. Unlike the previous Aperio AT2 DX System precision study where slide curation and study feature viewing/identification were performed at 20× and 40× magnification, the current study used only 40× magnification for FOV viewing/feature identification as the Aperio GT 450 DX can only scan at 40×.

The 69 slides generated 202 FOVs (with each slide or paired scanned image having one–three histological features). Pathologists were blinded to the number of features present within each FOV. The slide panel and the tissue features were used from the previous FDA-cleared AT2 DX System study^8^ (510k number K190332). Table 1 details the panel information. To minimize study slides recall bias, an additional 12 wild card slides were also used (Table A.1) which were excluded from the eventual data compilation and analyses.

**Table 1:** Primary histological features selected in the 69-slide precision panel.

FOV magnification resolution	Feature	Specimen source/Organ type
20×	Chondrocytes	Toe
		Femoral head
		Osteosarcoma of humerus
	Fat cells (adipocytes)	Axillary lymph node
		Femoral head
		Prostate
	Foreign body giant cells	Knee synovium
		Shoulder
		Sigmoid colon
	Goblet cells in intestinal mucosa/intestinal metaplasia	Gastroesophageal junction
		Sigmoid colon
		Tubular adenoma (intestine)
	Granulomas	Colon
		Iliac crest (bone)
		Cervical lymph node
	Infiltrating or metastatic lobular carcinoma	Iliac crest (bone)
		Jejunum
		Breast
	Intraluminal necrosis	Lung
		Liver
		Right colon
	Osteoclasts	Sacrum
		Toe
		Paget's disease of spine
	Osteocytes	Foot
		Maxilla
		Osteosarcoma of femur
	Pleomorphic nucleus of malignant cell	11th rib
		Sacrum
		Vertebra
	Serrated intestinal epithelium (for example, sessile serrated lesion/adenoma)	Appendix
		Ascending colon polyp
		Sigmoid colon
	Skeletal muscle fibers	Lower leg
		Shoulder
		Spine
40×	Asteroid bodies	Axillary lymph node
		Liver
		Synovium
	Clear cells (renal cell carcinoma)	Humerus
		Retroperitoneal lymph node
		Right kidney
	Foreign bodies (for example, plant material or foreign debris)	Distal femur
		Foot
		Wrist
	Hemosiderin (pigment)	Knee synovium
		Liver
		Osteosarcoma of femur
	Megakaryocytes	Cervical spine
		Femur (margin of sarcoma)
		Tibia
	Necrosis	Femoral head
		Para-aortic lymph node
		Right leg
	Nerve cell bodies (for example ganglion cells)	Ganglioneuroma
		Small bowel
		Stomach
	Nuclear grooves	Cervical lymph node (papillary thyroid carcinoma)
		Iliac crest (bone) (Langerhan's cell granuloma)
		Ovary (Brenner tumor)
	Osteoid matrix	Femur
		Humerus
		Lung
	Psammoma bodies	Cervical lymph node (metastatic papillary carcinoma of thyroid)
		Fallopian tube (papillary ovarian carcinoma)
		Left ventral cranial region (meningioma)
	Reed–Sternberg cell	Axillary lymph node
		Neck mass
		Spleen

### Study design

The precision of the Aperio GT 450 DX in identifying histological features important for diagnoses was evaluated in three sub-studies: intra-system, inter-system/site, and inter-/intra-pathologist. The assessments were based on the pathologists' identification (i.e., reading) of select histological features (e.g., chondrocytes, fat cells) observed in WSIs created from scanning a set of 69 FFPE H&E-stained tissue slides (precision panel) on the Aperio GT 450 DX. The hisotechnician scanned glass slides at 40× magnification, whereas the study pathologists viewed the extracted FOVs from each WSI at 20× or 40× magnification to replicate conventional microscopy magnifications used to identify selected features in a clinical setting.

The study inclusion criterion required glass slide mounted FFPE tissue samples stained with H&E containing the pre-defined study features. Slides were excluded (exclusion criteria) from the study if they were broken or had unremovable marks, or the H&E staining was washed-out and significantly influenced feature identification. The precision study enrolled 69 slides to analyze device precision, divided into groups of 23 each across each of the three sites for the intra-system study. For inter-system/site analyses, the full set of 69 slides was scanned by technicians at each of the three sites, whereas for inter−/intra-pathologist sub-study 69-slide panel was scanned at only one site.

The slides were read by five board-certified reading pathologists across three investigational sites (a fourth site was used to rescan four slides that were initially scanned at site 2; Supplementary Table A.2). The same curated slides used in the Aperio AT2 DX System precision study^8^ were also used in the current study. The case curation and annotation, which were performed in the previous Aperio AT2 DX System study,^8^ were not performed at any of the study sites so that the reading pathologists could be blinded to the annotated FOVs of the associated cases.

IRB approval of the protocol was obtained before the study initiation. Instead of a unique case read or diagnosis, the study included the identification of 23 unique primary histological features described in Table 1. The absence or presence of a selected feature was recorded in a secure eCRF (electronic case report form) by the study pathologist, which was shared with the biostatistician to evaluate the agreement rates across studies.

### Sub-studies

The same 69-slide panel was used in the current study to perform precision analyses that was used in the previous Aperio AT2 DX System precision study.^8^ The extracted features and the corresponding FOVs in each of the sub-studies is provided in Table 2.

**Table 2:** Whole slide images, field of views, and features identified across each study.

Precision study	WSIs	FOVs	Features
*Intra-system*			
System 1	69	201	261
System 2	69	201	276
System 3	69	204	222
Total	207	606	759

*Inter-system/Site*			
System 1	69	202	253
System 2	69	202	253
System 3	69	202	253
Total	207	606	759

*Intra- and inter-pathologist*			
Pathologist 1	69	202*	253
Pathologist 2		202*	253
Pathologist 3		202*	253
Total	69	606	759

Note:* Each extracted FOV was saved in three different orientations (202 FOVs × 3 orientations = 606 FOVs). Each of the three pathologists evaluated all FOVs in all orientations (i.e., 606 FOVs per pathologist).

Three sites (each having one Aperio GT 450 DX) were selected to perform the slide scanning and validation studies. Additionally, five study pathologists (three pathologists for intra- and inter-pathologist studies, and one each for intra- and inter-system/site studies) read the digital images.

### Intra-system study

**Fig. 2:**
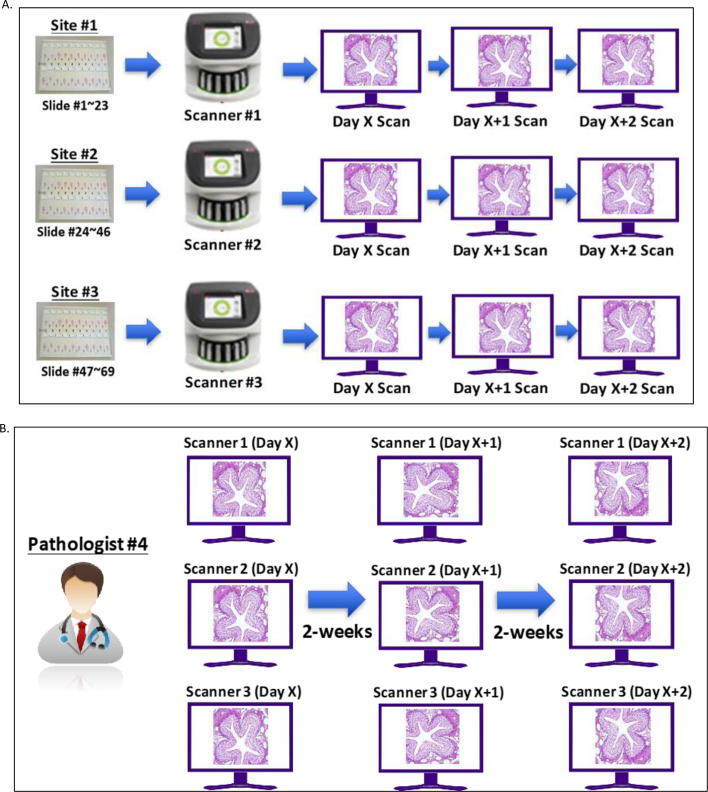
Schematic depiction of intra-system study workflow. (A) Scanning schema. The 69 precision panel slides were split equally across each system (i.e., 23 slides per system). The same 23 slides were scanned once on each of the 3 days for each system. The same process was followed for the three systems across three sites (B) Reading and analysis schema. All the FOVs generated by the corresponding WSI for each system/site were sent by the technician to the pathologist for reading and analysis. After a minimum 14-day washout period, the other slide subset scanned on the second day was read and analyzed by the pathologist. The same process was followed for FOVs generated on Day 3. In summary, FOVs from the set of 69 slides were read in each of the three reading sessions, with a washout period in between reading sessions.

Intra-system precision was conducted at three sites, each site with a single Aperio GT 450 DX scanner. The precision panel of 69 slides was split equally across three sites (23 slides × 3 sites). The scanning process was undertaken over 3 days (Fig. 2A); at each site, 23 slides were scanned once on each of the 3 days, producing three sets of WSIs for each slide, totaling 69 WSIs (23 slides × 3 days) per system. Across three sites, a total of 207 WSIs (69 WSIs × 3 systems) were generated. From the 207 WSIs, 606 FOVs (201 for systems 1 and 2 each, whereas 204 for system 3) were generated and sent to the single study pathologist for review (Fig. 2B). Of the 606 FOVs, 759 histological features (261 for system 1, 276 for system 2, and 222 for system 3) were reviewed. FOVs from the set of 69 slides were read on each of the three reading sessions, with a washout period of >14 days between reading sessions, as recommended by CAP guidelines.^9^ There was a different FOV orientation angle in each of the three reading sessions (Fig. 2B). Once the pathologist (pathologist #4) recorded the observation(s) in the eCRF, these were then sent to the statistician to interpret the findings and the associated agreement rate.

### Inter-system/site study

**Fig. 3:**
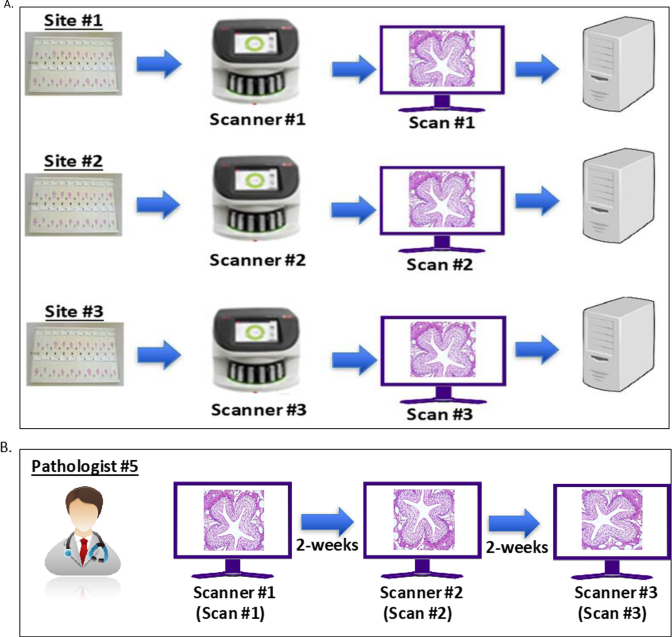
Schematic depiction of inter-system study workflow. (A) Scanning schema. A total of 69 slides were scanned by each system. The same panel of 69 slides was scanned by the technicians in a single day for each system across three sites. (B) Reading and analysis schema. All the FOVs generated by the corresponding scanned WSI for each system/site were sent by the technician to the pathologist for reading and analysis. Initially, FOVs scanned on the first scanner were read. After a minimum of 14 days gap (washout period), a second set of FOVs having different orientation angle scanned on second scanner were read and analyzed by the same pathologist. The same process was followed for a third set of FOVs generated on the third scanner at an orientation angle different from the FOVs of the other two scanners.

The panel of 69 slides was scanned once at each site (Fig. 3A). Across each of the three sites, FOVs were extracted from the WSI by the study technician and forwarded to the study pathologist for feature identification. All the FOVs were shared with the pathologist, who completed the reading of each slide panel in each session, completing three sets (triplicate of 69 slides for each site/system), with a washout period of >14 days between each of the three reading sessions (Fig. 3B). For each of the three reading sessions, the selected FOV was stored in a different orientation angle (Fig. 2B). Each system scanned 202 FOVs, with 253 identified features, totaling 606 FOVs (202 FOVs × 3 systems) and 759 features (253 features/system × 3 systems). After the observations were recorded in the eCRF by the reader (pathologist #5), these were shared with the study statistician for data analyses.

### Intra- and inter-pathologist study

**Fig. 4:**
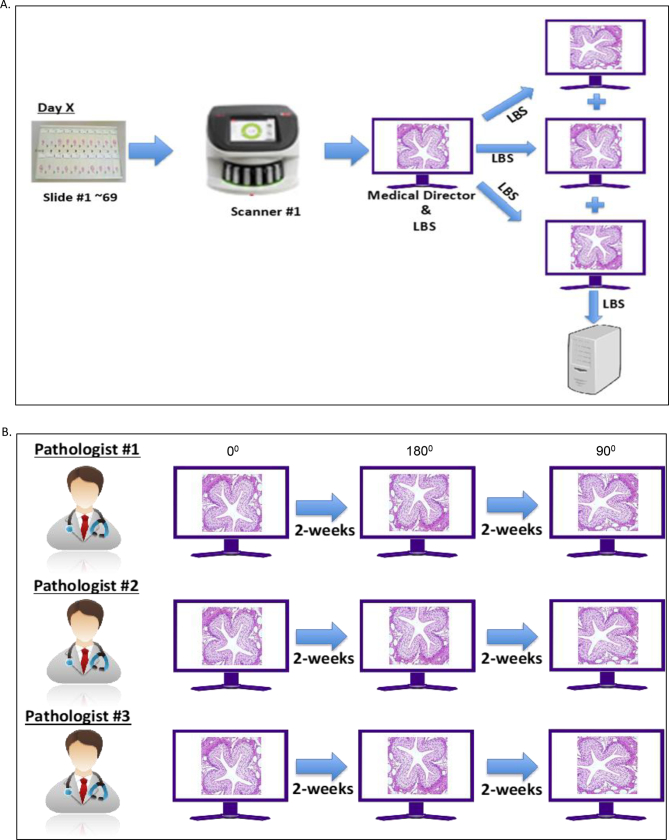
(A) Intra- and inter-pathologist scanning schema: A technician scanned the 69-panel slides and stored in three different orientations at a single site, which was shared with the three pathologists for FOV read and analyses (B) Reading schema of intra- and inter-pathologist analysis. A single day analysis included three reading sessions performed by each of the three study pathologists reading the 69 slides/extracted FOVs in the same orientation. After a minimum 14-day gap (washout period), pathologists read the same 69 slides in a different orientation, and the same process was followed for the third reading session following a minimum of 14-day gap, which had a different orientation than the previous two. The orientations were randomly rotated clockwise to 0 (original position), 90, 180, or 270 degrees relative to the original position. FOV, field of view; LBS, Leica Biosystems.

The 69-slide panel was scanned by the study technician at site #3. For each slide, FOV triplicates with the identified features were extracted. The FOVs were saved in three different orientations (Fig. 4A), with two FOVs being placed at an angle of either 90, 180, or 270 degrees and the other one being saved in its original position (0 degrees). In addition to the original position of the FOV, all the extracted FOVs with three different orientations were transferred to each of the three reading pathologists (Fig. 4B). Three sessions were performed to read the FOVs. In the first session, after a gap of >14 days, the same FOVs in a different orientation were read by all three pathologists. Lastly, after a gap or a washout period of >14 days, a third session was conducted by the pathologist to read the same FOVs in a third orientation. Agreement rates between orientation 1 vs orientation 2, orientation 2 vs orientation 3, and orientation 3 vs orientation 1 were evaluated for each pathologist across each reading session. The OA rate was based on the pooled analyses of the estimates.

For inter-pathologist analysis, feature identification agreement rates (absence or presence of the selected characteristic tissue features) were calculated across pathologists for each reading session. The agreement rates across pathologist 1 vs pathologist 2, pathologist 2 vs pathologist 1, and pathologist 2 vs pathologist 3, for each orientation were calculated. The overall inter-pathologist agreement rate was based on the pooled analyses of the estimates.

### Statistical analysis

The device precision was evaluated by comparing the agreement rate across the three sub-studies. The agreement rate CI was determined based on the 2.5th and 97.5th percentile of the bootstrap samples. If the agreement rate was 100%, then the 95% CI was calculated using the arcsine transformation and continuity correction approach. The percent agreement was calculated using the method below:

$$\text{Percent agreement}=\text{number of pairwise agreements}/{\text{number of total comparison pairs}}^{*}\;100.$$

The sample size of a 69-slide study panel was used from the previous Aperio AT2 DX System precision study,^8^ and hence, sample re-estimation was done for the Aperio GT 450 DX precision study. The study FOVs, its features, the FOV rotation angle (0°, 90°, 180°, or 270°), and the annotation color as deidentified and randomized by an independent biostatistician who did not participate in the study, and the study pathologists were blinded to the original identity of the FOVs and its features. During the final data analysis, this randomization list was provided to the study biostatistician to calculate the agreement rates for each sub-study.

All the analyses were completed using the SAS statistical software (v9.4).

## Results

### Intra-system study analyses

A set of 69 slides was split into 23 slides per each reading session for a system. Each reading session included FOVs extracted from 69 unique slides (23 unique slides/system × 3 systems) across three systems housed at three sites.

After a washout period of >14 days, sessions 2 and 3 were completed with a different orientation of the same FOV for each of the three reading sessions across systems. The FOV and the study features identified differed in number for each system. From each WSI, two or more unique FOVs were extracted, where each FOV could have one–three histological features, implying variability in extracted FOV and corresponding features for each WSI. Therefore, because the glass slides were assigned randomly—for each corresponding WSI—FOVs and the features were variable across systems.

The agreement rate for each system was calculated (87 features × 3 sessions for System 1 = 261 features; 92 features × 3 sessions for System 2 = 276 features; and 74 features × 3 sessions for System 3 = 222 features). An overall pooled intra-system agreement rate of 97.1% (737 agreements/759 total features) was calculated with a lower bound of 95% CI as 95.8% (Table 3), which met the predefined acceptance criteria of the lower bound of 95% CI being ≥85%.

**Table 3:** Intra-system analysis.

			Agreement rate and 95% CI		
System	Number of pairwise agreements	Number of comparison pairs	% Agreement	Lower	Upper
System 1	255	261	97.7	95.7%	99.3%
System 2	270	276	97.8	95.9%	99.3%
System 3	212	222	95.5	92.5%	98.1%
Overall	737	759	97.1	95.8%	98.3%

Note: 1. 95% CI was produced using the percentile bootstrapping approach on 5000 bootstrap samples. 2. System 1 was at site 1, System 2 was at site 2, and System 3 was at site 3.

The agreement pairs for each system were evaluated for session 1 vs session 2, session 2 vs session 3, and session 3 vs session 1. The most frequently unidentified features across systems were necrosis, asteroid bodies, and hemosiderin.

### Inter-system/site precision analyses

The 69-slide panel were scanned for each system. A single pathologist read 69 whole slides in one session, whereas the subsequent sessions (total of three) were conducted after a washout period of >14 days. To minimize recall bias, each FOV was scanned at three different orientations for each session. For each system, 202 FOVs were extracted with 253 features, totaling 759 features (253 features × 3 systems). An OA rate of 96.3% was observed, with a lower bound of 95% CI as 94.9% (Table 4), which met the predefined acceptance criteria of the lower bound of 95% CI being ≥85%.

**Table 4:** Inter-system/site precision analyses.

			Agreement rate and 95% CI		
System	Number of pairwise agreements	Number of comparison pairs	% Agreement	Lower	Upper
System 1 vs System 2	241	253	95.3	92.5%	97.7%
System 1 vs System 3	246	253	97.2	95.0%	99.2%
System 2 vs System 3	244	253	96.4	94.0%	98.5%
Overall	731	759	96.3	94.9%	97.6%

Note: 1. 95% CI was produced using the percentile bootstrapping approach on 5000 bootstrap samples. 2. System 1 was at site 1, System 2 was at site 2, and System 3 was at site 3.

Reed–Sternberg cell (40×), hemosiderin (40×), and osteoclasts (20×) were the most frequently unidentified features within a FOV.

### Intra- and inter- pathologist analyses

For intra-pathologist analysis, FOVs at three different orientations were read by each pathologist. A total of three pathologists read the digitized image. For each pathologist, agreement rates for orientation 1 vs orientation 2, orientation 2 vs orientation 3, and orientation 3 vs orientation 1 were evaluated. An agreement rate of a total of 606 FOV (202 FOV × 3 orientations) containing 759 features (253 features/reading session × 3 orientations) per pathologist was evaluated. An OA rate of 93.5% was calculated with a lower bound of 95% CI as 92.4% (Table 5), which met the predefined acceptance criteria of the lower bound of 95% CI being ≥85%.

**Table 5:** Intra-pathologist analyses.

			Agreement rate and 95% CI		
Pathologist	Number of pairwise agreements	Number of comparison pairs	% Agreement	Lower	Upper
Pathologist 1	729	759	96.0	94.7%	97.3%
Pathologist 2	677	759	89.2	86.8%	91.3%
Pathologist 3	723	759	95.3	93.7%	96.7%
Overall	2129	2277	93.5	92.4%	94.5%

Note: 1. 95% CI was produced using the percentile bootstrapping approach on 5000 bootstrap samples.

Fat cells (adipocytes) and hemosiderin were the two features that were not identified at least once by the three pathologists.

For inter-pathologist analyses, each pathologist read 69 slides and 606 FOV (202 FOVs × 3 orientations) totaling 2277 (253 features × 3 orientations × 3 pathologists) identified study features. A comparative analysis was done between pathologist 1 vs pathologist 2, pathologist 1 vs pathologist 3, and pathologist 2 vs pathologist 3. An overall inter-pathologist agreement of 91.7% was calculated with a lower bound of 95% CI as 90.6% (Table 6), which met the predefined acceptance criteria of the lower bound of 95% CI being ≥85%.

**Table 6:** Inter-pathologist analyses.

			Agreement rate and 95% CI		
Pathologist	Number of pairwise agreements	Number of comparison pairs	% Agreement	Lower	Upper
Pathologist 1 vs Pathologist 2	686	759	90.4	88.2%	92.4%
Pathologist 1 vs Pathologist 3	727	759	95.8	94.3%	97.2%
Pathologist 2 vs Pathologist 3	676	759	89.1	86.9%	91.2%
Overall	2089	2277	91.7	90.6%	92.8%

Note: 1. 95% CI was produced using the percentile bootstrapping approach on 5000 bootstrap samples.

Hemosiderin, pleomorphic cells of malignant cells, and fat cells (adipocytes) were the top primary features that were not identified by the pathologists.

### Discrepant features

For the pooled intra-system study, out of the 11 discordant estimates, the most discrepant reads were observed for necrosis (3/11), hemosiderin (2/11), and asteroid bodies (2/11). For the pooled inter-system/site, out of 19 discordant reads, Reed–Sternberg cell (4/19), hemosiderin (4/19), and osteoclasts (3/19), and pleomorphic nucleus of malignant cells (3/19) encumbered the most discordant results. For the pooled inter-pathologist study, out of the 94 discordant observations, pleomorphic nucleus of malignant cells (15/94), fat cells (adipocytes; 15/94), and hemosiderin (12/94) had most discordant results, whereas for pooled intra-pathologist analyses, out of the 81 discordant observations, fat cells (adipocytes; 15/81), and hemosiderin (12/81) were the commonly observed discordant features.

## Discussion

This study demonstrated that pathologists using the Aperio GT 450 DX WSI system can precisely detect histological features in FFPE tissue samples.

Many studies have demonstrated that using a WSI system is non-inferior to using traditional microscopy.^1^, ^2^, ^3^ However, few published studies^8^ have recorded the analytical precision of the WSI system by validating the repeatability and reproducibility of feature identifications necessary for the correct diagnosis. This is also the first study where the Aperio GT 450 DX, as a diagnostic aid, is comprehensively tested by performing inter- and intra-observer and inter- and intra-system analyses. Out of the four FDA-cleared WSI systems,^11^, ^12^, ^13^, ^14^ other than the current study, only the Aperio AT2 DX System study (Aperio GT 450 predecessor) has published^8^ validation of the WSI system's precision capabilities. Other DP imaging systems may have submitted complete analytical precision findings in their regulatory filings but, to our knowledge, have not been published in the peer-reviewed literature. Few studies have separately tested essential components such as inter−/intra-observer^15^^,^^16^ or inter/intra-system variability (using novel artificial intelligence algorithms),^17^ but barring the Aperio AT2 DX System study,^8^ none to our knowledge have published the WSI system's repeatability and reproducibility capabilities in a single non-interventional study.

The DPA has identified 23 features essential for the diagnosis of pathology cases. Therefore, we selected each of these morphological characteristic identifiers from three different organ types and extracted FOVs at 20× and 40× magnification. The current study used the same 69-panel slides, which were used to analyze the precision of the Aperio AT2 DX System precision study.^8^ To minimize study bias, pathologists were not given access to any diagnostic information described in the original sign-out report.

Inter-observer and inter-site/system variability is a common concern in anatomic pathology^18^^,^^19^ and can lead to delayed and sometimes erroneous patient management. The current study tested the precision of the Aperio GT 450 DX by analyzing both repeatability (intra-pathologist and intra-system) and reproducibility (inter-pathologist and inter-system) by conducting three studies and applying appropriate statistical tools to calculate the agreement rate and comparing it against predefined acceptance criteria. For all three tested components, the percent OA rate was well above the threshold acceptance criteria of ≥85%, indicating low inter/intra-observer and inter/intra-system/site variability.

**Fig. 5:**
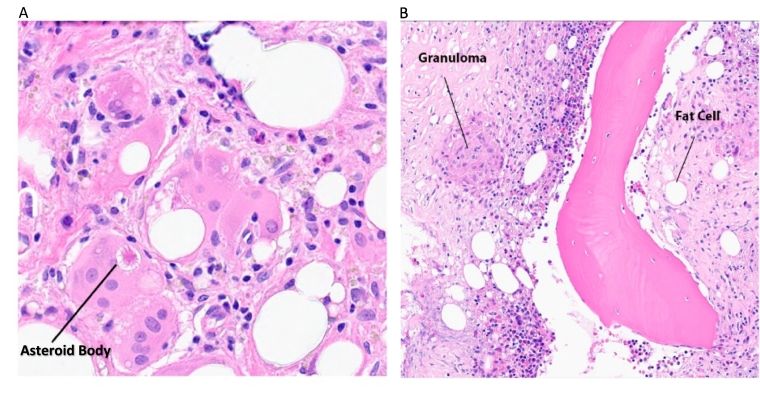
(A) The arrow indicates an asteroid body within the cytoplasm of a giant cell. (B) Although intended as an example of fat cells, the intended cell could have been interpreted as a vacuole or artifact, and viewers might have been distracted by the granuloma.

Discordances were observed in identifying the hemosiderin pigment across sub-studies. The reason for the high discrepancy rate for this specific feature is thought to be due to the absence of additional clinical information that is usually provided to the pathologist in routine clinical practice. Additionally, the slides were only H&E-stained and special staining request by the pathologist was not permissible for this study, in contrast to regular clinical practice where special stains to identify iron are commonly used.^20^ Variable subjectivity for inter-pathologist classification of hemosiderin is also a known issue.^20^ Another histomorphological feature that showed discrepancies in this study was detecting Reed–Sternberg cells. The morphology of Reed–Sternberg cells is variable, and in this study, no attempt was made to include only “classic” cases. There was variability in identifying these cells in this study, similar to rates documented in the literature compared with general pathologists who were not board-certified in hematopathology.^21^ Ancillary immunohistochemical staining, such as CD15 and CD30, which is commonly available in clinical practice was not provided in the current study. Necrosis is also one of the features which is subjective, the identification of which may differ from one pathologist to the other.^22^ Asteroid bodies, which was another feature with relatively high discordance, are relatively rare^23^; so pathologists may have had hesitation in confirming the morphology of this feature. The pleomorphic nucleus of malignant cells is a generic term, and a more inclusive/cancer type specific term/wording may have aided the pathologists in better identifying the said feature. Lastly, IHC stains such as CD13, CD31, or CD51 (beyond study scope) might have helped in improved identification of osteoclasts.^24^ Fig. 5 shows the representative micrographs of some of the discordant features.

The rescan rate for the Aperio GT 450 DX was 1.7% (6 rescanned WSIs/total 345 WSIs), which was within the recommended limit of <5% (see supplementary Table A.2).^25^ Additionally, any issue identified by the auto-quality control feature of the whole slide system was either resolved automatically or manually.

Secure acquisition of digital data and its integration in the hospital/academic center LIS is a key to the continuous adaption of digital pathology for routine clinical use.^26^ To some extent, the current study was able to address questions on the WSI system's secure data processing and demonstrated the system's potential for being used as a diagnostic aid by successfully executing detailed testing of the system's repeatability and reproducibility with low variability across pathologists and sites. This precision study demonstrated the ability of Aperio GT 450 DX to generate reproducible optimal quality images. Additionally, in another study, Aperio GT 450 DX's diagnostic accuracy was found to be non-inferior to glass slide reads (manuscript submitted).

The Aperio GT 450 DX has been comprehensively tested for diagnostic accuracy and precision. The fact that this digital pathology workflow is cleared by the FDA gives further evidence that the Aperio GT 450 DX WSI system can be useful for accurate diagnosis. Additionally, the pathologists easily met the acceptance criteria for each tested precision component even though the study included difficult features such as Reed–Sternberg cells, which were not of “textbook” quality and replicated real-world clinical setting, implying that this digital pathology workflow solution can generate optimal quality histological images.

## Conclusion

This precision study met all the predefined acceptance criteria for inter/intra-observer and inter-site/system components. These data show that pathologists using the Aperio GT 450 DX WSI system can precisely identify essential histological features that may be necessary for accurate diagnoses of anatomical pathology cases.

## Funding

This work was supported by the Leica Biosystems Imaging, Inc.

## Declaration of competing interest

Bauer is a consultant at Leica Biosystems Imaging, Inc. and Moximed, Inc., and Deputy Editor for Research-Journal of Bone and Joint Surgery; Zhou, Lewis, Dayal, Chiweshe, Ferber, Ergin Sutcu, and White are employees of Leica Biosystems Imaging, Inc.

## Appendix A Supplementary data

Supplementary material Tabulated Wild Card slide sources and list of slides that were rescanned. Supplementary material
