# Supplementary material for: A multicenter study to evaluate the analytical precision by pathologists using the Aperio GT 450 DX
Source: J Pathol Inform. 2024 Oct 9;15:100401. doi: 10.1016/j.jpi.2024.100401 (PMC11600015; doi:10.1016/j.jpi.2024.100401)
Supplement: Supplementary material — Tabulated Wild Card slide sources and list of slides that were rescanned. [file mmc1.docx]

**Supplemental data**

**Table A.1. Wild Card Slides from a range of tissue types**

| FOV Magnification  Resolution | Specimen Source/Organ Type |
| --- | --- |
| 20x | Liver |
|  | Adrenal |
|  | Salivary Gland |
|  | Skin |
|  | Colon |
|  | Respiratory mucosa, sinus content |
|  | Nerve |
|  | Cervix |
|  | Endometrium |
| 40x | Liver |
|  | Skin |
|  | Breast |

**Table A.2. Details of 6 slides that were rescanned**

| **System** | **.svs File Name** | **Case ID** | **Scan Run** | **Scan Date** | **QC Disposition** | **Rescan Reason** | **Rescan Date** | **Rescan Disposition** | **Description** |
| --- | --- | --- | --- | --- | --- | --- | --- | --- | --- |
| 2* | AS-LBS-018-1 | LBS143002 | 1 | 08 DEC 2021 | Not Acceptable | Poor image quality | 17FEB2022 | Acceptable | Scan is blurry and features are not well defined. |
| 2* |  | LBS439590 | 1 | 08 DEC 2021 | Not Acceptable | Poor image quality | 17FEB2022 | Acceptable | Scan is blurry and features are not well defined. |
| 2* |  | LBS784067 | 1 | 08 DEC 2021 | Not Acceptable | Poor image quality | 17FEB2022 | Acceptable | Scan is blurry and features are not well defined. |
| 2* | AS-LBS-048-1 | LBS351309 | 1 | 08 DEC 2021 | Not Acceptable | Poor image quality | 16MAR2022 | Acceptable | Left side of FOV is blurry. |
| 2* |  | LBS689925 | 1 | 08 DEC 2021 | Not Acceptable | Poor image quality | 16MAR2022 | Acceptable | Left side of FOV is blurry. |
| 2* |  | LBS785619 | 1 | 08 DEC 2021 | Not Acceptable | Poor image quality | 16MAR2022 | Acceptable | Left side of FOV is blurry. |
| 2* | AS-LBS-081-2 | LBS916242 | 1 | 09 DEC 2021 | Not Acceptable | Poor image quality | 16MAR2022 | Acceptable | Black smear obscures part of image. |
| 2* | AS-LBS-081-3 | LBS621671 | 1 | 09 DEC 2021 | Not Acceptable | Poor image quality | 16MAR2022 | Acceptable | Black smear obscures part of image. |
| 3 | AS-PRP-001-3 | LBS785414 | 1 | 28 APR 2022 | Not Acceptable | Poor image quality | 16-Jun-22 | Acceptable | Scan is blurry and features are not well defined. |
| 3 | AS-PRP-032-1 | LBS178029 | 1 | 25 APR 2022 | Not Acceptable | Poor image quality | 16-Jun-22 | Acceptable | Scan is blurry and features are not well defined. |
| 3 |  | LBS463675 | 1 | 25 APR 2022 | Not Acceptable | Poor image quality | 16-Jun-22 | Acceptable | Scan is blurry and features are not well defined. |
| **Note:**  1. System 1 is at site 1, System 2 is at site 2, and System 3 is at site 3  2. *Rescanning of slides was conducted on System 2 at site 4 | | | | | | | | | |
